# Post-vitrectomy endophthalmitis complicated with orbital cellulitis treated with hyperbaric oxygen therapy: A case report

**DOI:** 10.1097/MD.0000000000042043

**Published:** 2025-04-04

**Authors:** Yi-Ju Shen, Li-Ching Liu, I-Chia Liang

**Affiliations:** a Department of Ophthalmology, Tri-Service General Hospital, National Defense Medical Center, Taipei, Taiwan, (R.O.C); b Department of Ophthalmology, Cathay General Hospital, Taipei, Taiwan, (R.O.C).

**Keywords:** case report, endophthalmitis, hyperbaric oxygen therapy, silicon oil, vitrectomy

## Abstract

**Rationale:**

Infectious endophthalmitis developed in silicone oil (SO)-filled eyes after pars plana vitrectomy is a very rare but serious complication. Hyperbaric oxygen therapy (HBOT) has been reported to be effective in few studies of infectious endophthalmitis and orbital cellulitis.

**Patient concerns:**

This study reported a case of 71-year-old female with swelling of her left eye 1 week after pars plana vitrectomy and SO tamponade.

**Diagnoses:**

Acute infectious endophthalmitis complicated with orbital cellulitis and compartment syndrome in a postvitrectomy SO-filled eye.

**Interventions:**

Intravitreal injection of antibiotics and HBOT.

**Outcomes:**

The severe inflammatory condition of soft tissue with compartment syndrome and elevated intraocular pressure which could not be controlled ameliorated markedly after initiation of HBOT.

**Lessons:**

By taking advantages of the features of HBOT, it may be a good adjuvant treatment option for endophthalmitis cases along with antibiotics and surgical therapy.

## 1. Introduction

Endophthalmitis is a serious intraocular infection that can occur after various eye surgeries, including vitrectomy, though it is rare and challenging to manage.^[1–4]^ Advances in instrumentation and surgical techniques have reduced its incidence to between 0.02% and 0.13%.^[5–9]^ It is even rarer in silicone oil (SO)-filled eyes, likely due to the antimicrobial properties of SO.^[5,6,10]^ Treatment options for SO-filled post-vitrectomy endophthalmitis include intravitreal antibiotic injections alone or combined with surgical removal and reinjection of the SO.^[5,6,11,12]^

Hyperbaric oxygen therapy (HBOT) has been applied in ophthalmology^[13]^ during these decades and has been reported to be effective in few studies of infectious endophthalmitis and orbital cellulitis.^[14,15]^ Currently, no studies have reported the effects of HBOT in treating endophthalmitis with orbital cellulitis in post-vitrectomy SO-filled eyes. Here, we present a rare case of endophthalmitis complicated by orbital cellulitis after vitrectomy with SO infusion, treated with intravitreal antibiotics and HBOT.

## 2. Case report

**Figure 1.**
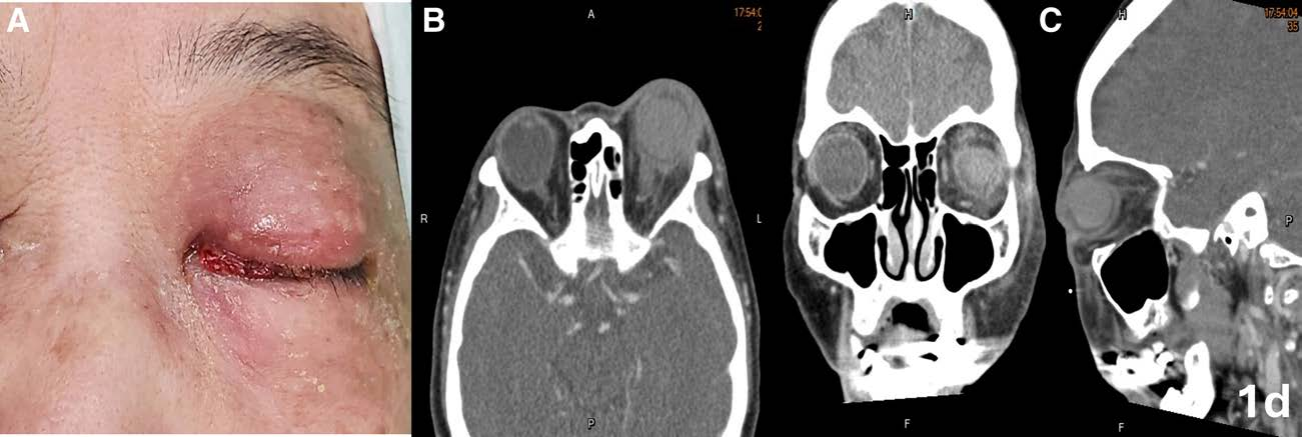
Initial presentation: (A) Severe eyelid swelling and firmness, preventing the eye from opening. (B–D) Axial, coronal, and sagittal CT scans show significant proptosis, hyperdensity in the vitreous and intraorbital fat stranding.

**Figure 2.**
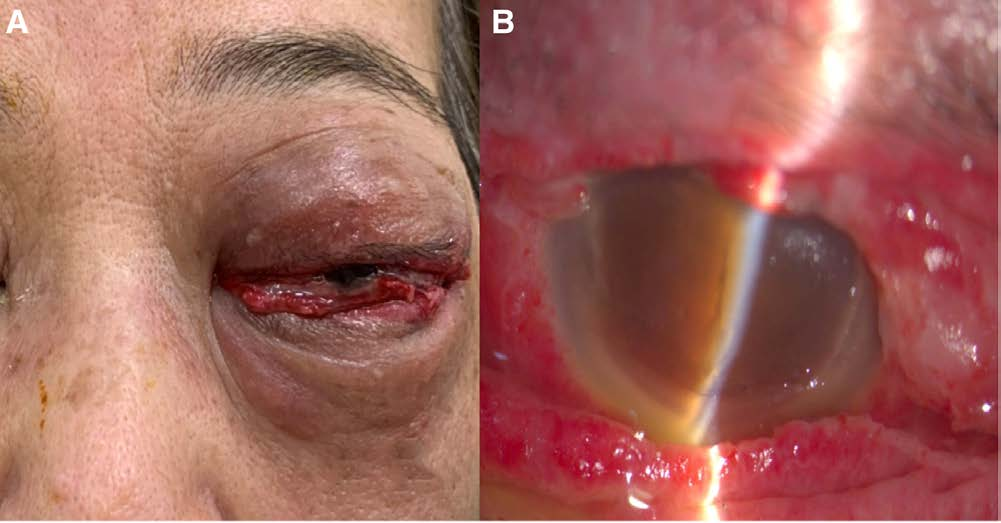
One day post-decompression: (A) Lagophthalmos with firm, swollen, and bruised eyelids. (B) Slit-lamp exam shows corneal edema and a hazy anterior chamber.

**Figure 3.**
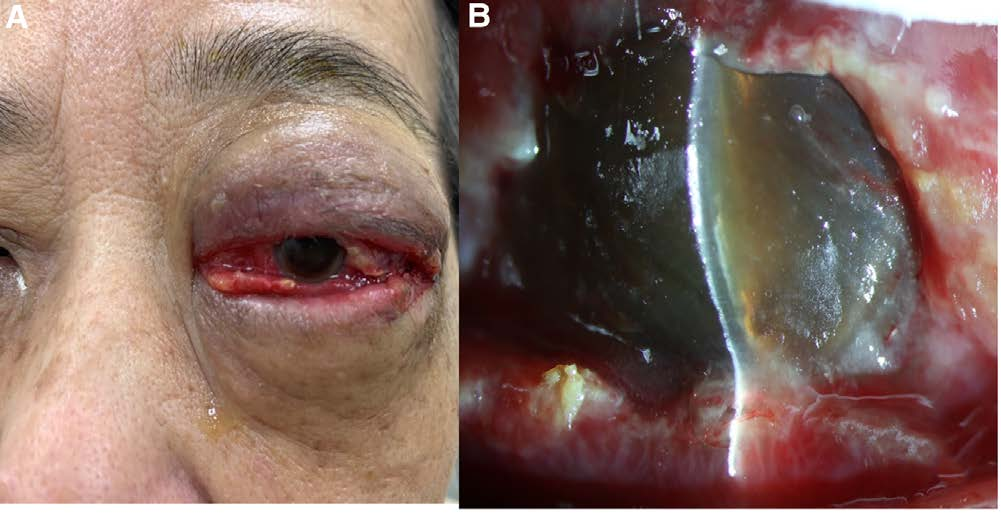
After 2 sessions of hyperbaric oxygen therapy: (A) Soft tissue swelling improved, with softer eyelids. (B) Slit-lamp exam shows reduced corneal edema and a clearer anterior chamber.

**Figure 4.**
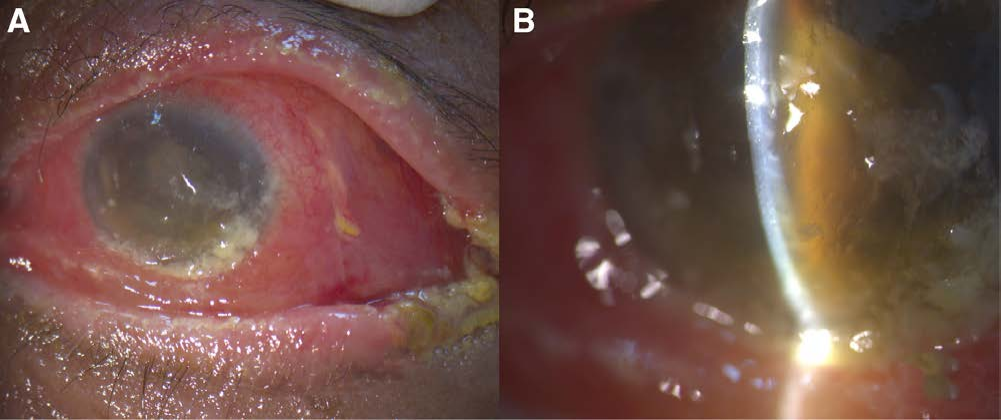
After 10 sessions of hyperbaric oxygen therapy: (A) Significant reduction in swelling. (B) Slit-lamp exam reveals a much clearer cornea and anterior chamber.

Informed consent was obtained from the patient for publication of this case report details, Institutional Review Board approval was obtained, and the report follows the principles of the Declaration of Helsinki. This 71-year-old woman with no systemic disease history experienced swelling of her left eye, headache, and nausea 1 week after undergoing surgery for foveoschisis and macular hole retinal detachment, which included pars plana vitrectomy and SO tamponade. As shown in Figure 1, her ophthalmological condition was difficult to assess due to severe eyelid swelling, and her visual acuity was no light perception. Orbital CT confirmed endophthalmitis with severe periorbital inflammation. Suspected acute endophthalmitis with orbital cellulitis and orbital compartment syndrome, the patient underwent emergent canthotomy, cantholysis, septum opening, and anterior chamber tapping for decompression. Intravitreal injections of ceftazidime (2.25 mg/0.1 mL), vancomycin (1 mg/0.1 mL), and subconjunctival injections of vancomycin (1 mg/0.1 mL), ceftazidime (2.25 mg/0.1 mL), and betamethason (0.4 mg/0.1 mL) were administered. Systemic antibiotics, including intravenous nemonoxacin (0.5g q24hr iv) and ceftazidime (2g q12hr iv), along with topical antibiotics (moxifloxacin eye drops 0.5% q2hr and tetracycline ointment 1% qid), were given. After 24 hours (Fig. 2), visual acuity remained no light perception, intraocular pressure was high (33.6 mm Hg), and swelling showed limited improvement. After ruling out contraindications and consulting with the patient and family, HBOT (2.5 ATA, 100 minutes daily) was promptly initiated. After 2 sessions, eyelid swelling improved significantly, and intraocular pressure dropped to 26.4 mm Hg (Fig. 3). Despite extensive infection tests, all cultures returned negative. The patient was discharged with improved visual acuity (light perception) and normalized intraocular pressure (10 mm Hg) after 10 courses of HBOT and continued antibiotic therapy (Fig. 4).

All the interventions were smoothly done according to the progress note and medical record. The patient deemed the therapeutic plan appropriate and effective to relieve her symptoms, and the prognosis was acceptable.

## 3. Discussion

Postoperative endophthalmitis is rare in post-vitrectomy cases, especially in eyes with SO tamponade.^[5,8]^ Additionally, the positive culture rate for post-vitrectomy endophthalmitis is very low,^[5,9,16]^ with only a few reported cases.^[12,17–23]^ This may be due to SO antimicrobial properties, including its bactericidal and fungistatic effects.^[6,10]^ Possible mechanisms include nutritional deprivation, toxicity from impurities, and compartmentalization that limits pathogen activity.^[24–27]^ Several cases of SO-filled endophthalmitis have been successfully treated with intravitreal antibiotics without removing the SO,^[11,12]^ leveraging its protective properties. This may also explain why our case showed infection signs but no positive culture result.

HBOT delivers pure oxygen at increased pressure, creating hyperoxemia and hyperoxia, which have been widely used in clinical conditions such as decompression sickness and carbon monoxide poisoning. HBOT has also been shown to accelerate wound healing and provide antimicrobial effects.^[28–30]^ It is particularly beneficial for tissues with poor perfusion, such as diabetic foot ulcers, compromised skin grafts, and crush injuries. HBOT promotes wound healing through vasoconstriction, enhanced angiogenesis, and collagen synthesis.^[28]^ Vasoconstriction reduces tissue edema by lowering pressure on peripheral vessels, improving perfusion for nutrient transport and waste removal.^[28,30]^ In addition, HBOT promotes vessel growth, which speeds up tissue repair. Angiogenesis in HBOT patients is marked by increased levels of factors such as endothelial growth factor, vascular endothelial growth factor, hypoxia-inducible factor 1-alpha, platelet-derived growth factor, fibroblast growth factor-2, and others like nitric oxide.^[28,30,31]^ The expression of the nuclear factor erythroid 2-related factor-2 gene, which regulates growth-promoting, antioxidant, and detoxifying enzymes, is also upregulated, further enhancing the healing process.^[32]^ Case reports have shown HBOT efficacy in wound healing and reducing inflammation in conditions like pseudomonas endophthalmitis and orbital cellulitis,^[14]^ as well as its bactericidal effects in orbital necrotizing fasciitis.^[33]^ HBOT antimicrobial effects are both direct, via oxidative stress, and indirect, through immunomodulation.^[34]^ Reactive oxygen species generated during HBOT inhibit anaerobic infections, as many pathogens lack antioxidant defenses like superoxide dismutase. Reactive oxygen species also cause cytotoxicity by damaging deoxyribonucleic acid, ribonucleic acid, proteins, and lipids, disrupting cell membranes, and interfering with protein functions.^[29]^ HBOT is often used alongside antibiotics, with a synergistic effect reported for certain antibiotics like β-lactams, quinolones, and aminoglycosides against aerobic organisms. Reoxygenation of infected tissues restores bacterial aerobic metabolism, improving the efficacy of these antibiotics.^[28,35]^ HBOT immunomodulatory effects include reducing inflammation by downregulating pro-inflammatory cytokines, promoting apoptosis of neutrophils and lymphocytes, and enhancing phagocytosis and neutrophil mobility via nitric oxide.^[29,30]^ These properties allow HBOT to target both aerobic and anaerobic pathogens, making it particularly useful in low culture rate infections like ours. In this case, the significant improvement in soft tissue likely resulted from the combined effects of decompression, immunomodulation, and HBOT antimicrobial properties. The strengths of this case report that there is no previous paper discussed about the advantages of HBOT in case of endophthalmitis with orbital cellulitis in post-vitrectomy SO-filled eyes. The limitations of this case report refer to the impossibility of establishing a cause–effect relationship, the retrospective study design, and the focus on an unusual case.

## 4. Conclusion

Infectious endophthalmitis in SO-filled eyes after pars plana vitrectomy is a rare but serious complication, with few reported cases. Concurrent severe periorbital cellulitis is even rarer. Negative culture results are common due to SO antimicrobial properties. HBOT, combined with antibiotics and surgery, could be a valuable adjuvant treatment for such cases.

## Author contributions

**Conceptualization:** Yi-Ju Shen, I-Chia Liang.

**Data curation:** Yi-Ju Shen, Li-Ching Liu.

**Formal analysis:** Yi-Ju Shen, Li-Ching Liu.

**Investigation:** Yi-Ju Shen, Li-Ching Liu.

**Methodology:** Yi-Ju Shen, Li-Ching Liu, I-Chia Liang.

**Project administration:** Yi-Ju Shen, I-Chia Liang.

**Software:** Yi-Ju Shen.

**Supervision:** I-Chia Liang.

**Validation:** I-Chia Liang.

**Visualization:** Yi-Ju Shen.

**Writing – original draft:** Yi-Ju Shen.

**Writing – review & editing:** I-Chia Liang.
